# A Novel Autosomal Recessive Variant of the *NRL* Gene Causing Enhanced S-Cone Syndrome: A Morpho-Functional Analysis of Two Unrelated Pediatric Patients

**DOI:** 10.3390/diagnostics12092183

**Published:** 2022-09-09

**Authors:** Giancarlo Iarossi, Lorenzo Sinibaldi, Chiara Passarelli, Andrea Maria Coppe’, Alessandro Cappelli, Gianni Petrocelli, Gino Catena, Chiara Perrone, Benedetto Falsini, Antonio Novelli, Andrea Bartuli, Luca Buzzonetti

**Affiliations:** 1 Department of Ophthalmology, Bambino Gesù Children’s Hospital, 00165 Rome, Italy; 2 Laboratory of Medical Genetics, Translational Cytogenomics Research Unit, Bambino Gesù Children’s Hospital, 00146 Rome, Italy; 3 Rare Disease and Medical Genetics, Bambino Gesù Children’s Hospital, 00146 Rome, Italy

**Keywords:** enhanced S-cone syndrome, autosomal recessive inheritance, *NRL*, retinal degeneration, phenotype variability, specialized ERG response

## Abstract

Enhanced S-cone syndrome (ESCS) is a rare autosomal recessive retinal degeneration mainly associated with pathogenic variations in the *NR2E3* gene. Only a few pathogenic variations in the *NRL* gene associated with ESCS have been reported to date. Here, we describe the clinical and genetic findings of two unrelated pediatric patients with a novel frameshift homozygous variant in the *NRL* gene. Fundus examinations showed signs of peripheral degeneration in both patients, more severe in Proband 2, with relative sparing of the macular area. Spectral domain optical coherence tomography (SD-OCT) revealed a significant macular involvement with cysts in Proband 1, and minimal foveal alteration with peripheral retina involvement in Proband 2. Visual acuity was abnormal in both patients, but more severely affected in Proband 1 than Proband 2. The electroretinogram recordings showed reduced scotopic, mixed and single flash cone responses, with a typical supernormal S-cone response, meeting the criteria for a clinical diagnosis of ESCS in both patients. The present report expands the clinical and genetic spectrum of *NRL*-associated ESCS, and confirms the age-independent variability of phenotypic presentation already described in the *NR2E3*-associated ESCS.

## 1. Introduction

Enhanced S-cone syndrome (ESCS) is a rare autosomal recessive retinal degeneration characterized by night blindness, variable visual dysfunction, visual field alteration, and increased sensitivity to blue light. Typical features are nummular pigmentary deposits at the level of the retinal pigment epithelium outside the vascular arcades, frequently associated with macular schisis and dot-like lesions [1,2,3]. An electroretinogram (ERG) presents pathognomonic responses consisting of the absence of rod response, reduced M- and L-cone responses, and enhanced S-cone response. ESCS is mainly caused by pathogenic variations in the *NR2E3* gene, which encodes a rod-specific ligand-dependent transcription factor that plays a key role in rod differentiation [4] and suppresses S-cone opsins. The *NRL* gene encodes neural retina leucine zipper factor, which interacts with other transcription factors, and plays essential roles in the normal development of photoreceptor cells, regulating the activity of rod-specific genes and guiding a postmitotic photoreceptor into the rod lineage [5,6,7]. More specifically, the *NRL* gene engenders retinal orphan photoreceptor-specific nuclear receptor NR2E3, which together with NRL, induce rod genes and suppresses cone genes. Consequently, *Nrl-* and *Nr2e3*-knockout mice have no rod photoreceptors, but have a large excess of S-cone-like photoreceptors [8]. Autosomal dominant missense variations in the *NRL* gene have been reported to cause retinitis pigmentosa (RP) [9,10,11,12,13], while autosomal recessive variations of the *NRL* gene have been described, so far, in only eight patients belonging to six unrelated families. In detail, seven patients were associated with a clinical phenotype of ESCS and one patient was associated with an autosomal recessive retinitis pigmentosa (arRP). Nishiguchi et al. first reported on two siblings with compound heterozygous *NRL* variations, who presented a clinical phenotype indicative of ESCS [14]. Newman et al. described two unrelated patients affected by oculopharyngeal muscular dystrophy (OPMD) and retinal dystrophy with a ESCS-like phenotype caused by the same homozygous loss-of-function variation in the *NRL* gene [15]. Four other patients carrying autosomal recessive *NRL* pathogenic variants and different phenotypes have been detected by studying large groups of presumed arRP patients using high-resolution genome-wide homozygosity mapping, targeted NGS, and whole exome sequencing [16,17,18,19]. Extensive re-analysis of the phenotype confirmed *NRL* as a putative genetic cause of clinical ESCS in three cases and of arRP in the other case. In this work, we identified a novel autosomal recessive pathogenic change in the *NRL* gene in two unrelated pediatric patients and described, in detail, the clinical phenotype leading us to the diagnosis of ESCS.

## 2. Materials and Methods

### 2.1. Patient Studies, Clinical and Ophthalmological Examinations

All procedures in this study adhered to the tenets of the Declaration of Helsinki and were approved by the Ethic Committee of the Bambino Gesù Children’s Hospital (code 558/2012). Appropriate informed consent was obtained from the parents of the patients. Probands and their families underwent comprehensive age-appropriate ophthalmic examinations, which included best-corrected visual acuity (BCVA) measurements with the Early Treatment Diabetic Retinopathy Study (ETDRS) charts, expressed as a logarithm of the minimum angle of resolution (logMAR); slit-lamp biomicroscopy; indirect ophthalmoscopy with 20D lens and biomicroscopy with 90D lens (Volk); color fundus photos and blue light fundus autofluorescence (FAF) (Daytona wide-field retinography); spectral domain optical coherence tomography (SD-OCT) (Heidelberg Spectralis HRA+OCT, Heidelberg Engineering, Heidelberg, Germany); and a full-field electroretinogram (ERG) recorded according to the ISCEV standards (retimax recording system, CSO, Florence, Italy). In addition, specialized S-cone ERGs were recorded by using chromatic stimuli, as previously described by [20]. Briefly, S-cone ERGs were recorded presenting a blue (420 nm) Ganzfeld stimulus on a yellow background, whereas ML-cone ERGs were obtained presenting a red (580 nm) stimulus on a green background.

### 2.2. Molecular Genetic Study

DNA was extracted from peripheral blood using Qiagen columns (QIAamp DNA Mini kit, Qiagen, Hilden, Germany), according to the manufacturer’s instructions. The concentration and purity of the DNA samples were quantified using an ND-1000 spectrophotometer (NanoDrop, Thermo Scientific, Waltham, MA, USA) and an FLx800 Fluorescence Reader (BioTek, Winooski, VT, USA). A clinical exome (Twist Bioscience, South S. Francisco, CA, USA), using a custom panel including 6920 genes known as associated with genetic disorders, was performed on probands’ and parents’ genomic DNA, according to the manufacturer’s protocol, and sequenced on an Illumina NovaSeq6000 platform. The reads were aligned to human genome build GRCh37/UCSC hg19. The BaseSpace pipeline and the Geneyx software LifeMap Sciences were used for the variant calling and annotating variants, respectively. The variants were filtered by in silico analysis on genes associated with retinal dystrophies, considering the presence of all suspected variants in the public databases (dbSNP, Exome Aggregation Consortium (ExAC), and Genome Aggregation Database (gnomAD)) and the prediction of deleterious non-synonymous SNVs for human diseases. The global minor allele frequencies (MAF) for the analyzed variants were calculated according to the Genome Aggregation Database (gnomAD). The variants were evaluated using VarSome [21] and, for the purposes of the diagnostic classification and in accordance with the American College of Medical Genetics and Genomics (ACMG) recommendations [22], only variants categorized as likely pathogenic (level 4) or pathogenic (level 5) were considered. Variants were also examined for coverage and Qscore (minimum threshold of 30), and visualized using an Integrative Genome Viewer (IGV). The NRL variant was confirmed in the patients and in their parents by using Sanger sequencing, following a standard protocol.

## 3. Results

### 3.1. Patients’ Clinical Reports

**Figure 1:**
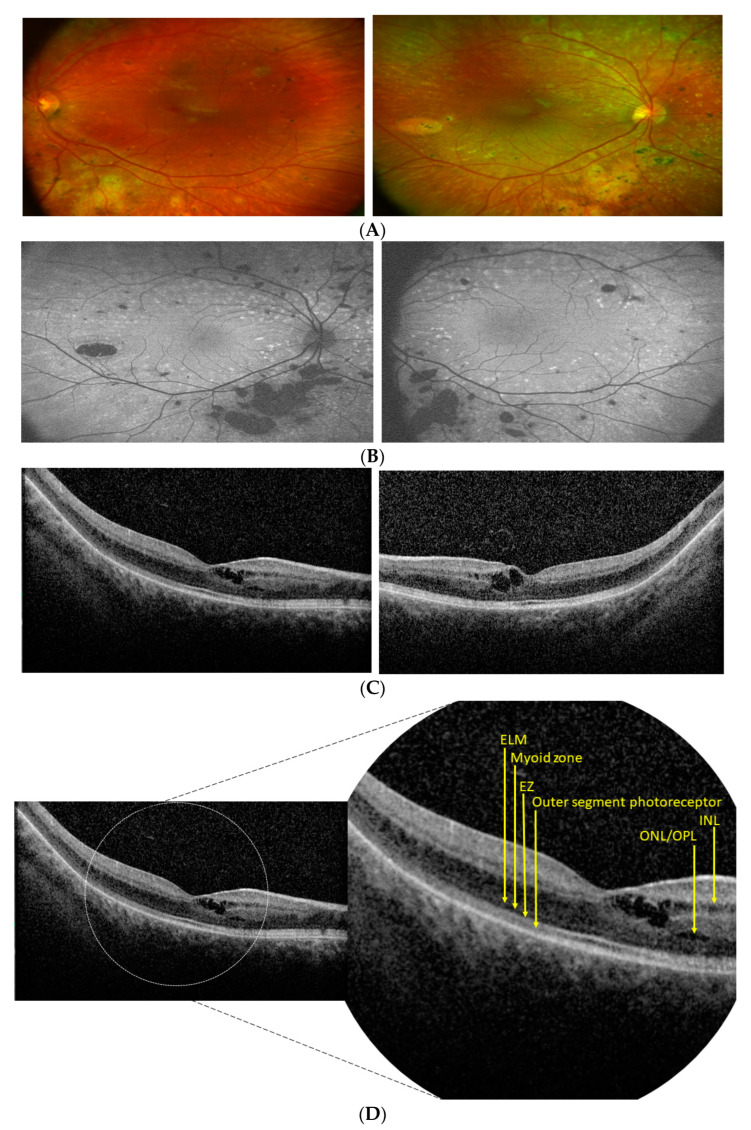
Retinal features of Patient 1: (**A**) Fundus images of the right and left eye showing areas of chorioretinal atrophy with yellowish dots and retinal pigment epithelium mottling at the posterior pole, more pronounced along the vascular arcades; (**B**) FAF images showing hypoautofluorescent patches corresponding to the atrophic lesions and hyperautofluorescent spots corresponding to the yellow dots; (**C**) HD SD-OCT macular B-scans showing a focal thickening of the fovea, associated with intraretinal lacunae of different sizes, greater in the central fovea and in the ONL and OPL. Microcysts are present in the INL. The contiguous ELM, the myoid zone, the EZ, and the outer segment photoreceptor layers are focally fragmented and present multiple areas of irregular optical density; (**D**) magnified image of the right eye HD SD-OCT macular B-scan showing, in detail, the different retinal layers: ELM (external limiting membrane), the myoid zone, the EZ (ellipsoid), outer segment photoreceptor layers, ONL/OPL (outer nuclear layer/outer plexiform layer), INL (inner nuclear layer).

Proband 1 was a female patient, aged 14 at the time of our first observation. Her parents were first cousins and the family history was unremarkable for relevant ocular disorders. The parents reported that she presented photophobia and nystagmus, relatively improved over time, and low vision since early childhood. At first functional examination, at the age of 4, her visual acuity had been reported as 0.3 LogMAR with a refractive error of +5.00 sph and +3 cyl/10 in the right eye (RE) and 0.22 LogMAR with a refractive error of +5.00 sph and +3.50 cyl/160 in the left eye (LE), whereas, at our observation, it was 0.45 LogMAR in the RE and 0.39 LogMAR in the LE, with a similar refractive defect. A fundus examination showed a normal optic disk, regular vessel caliber, and areas of chorioretinal atrophy with yellowish dots and retinal pigment epithelium mottling at the posterior pole, more pronounced along the vascular arcades, and relatively preserved macular area. High-definition (HD) SD-OCT macular B-scans showed a focal thickening of the fovea, associated with intraretinal lacunae of different sizes, greater in the central fovea and in the outer nuclear (ONL) and outer plexiform (OPL) layers, and the presence of microcysts in the inner nuclear layer (INL). The contiguous external limiting membrane (ELM), the myoid zone, the ellipsoid (EZ), and the outer segment photoreceptor layers were focally fragmented and presented multiple areas of irregular optical density. FAF showed hypoautofluorescent patches corresponding to atrophic lesions, with hyperautofluorescent spots corresponding to yellow dots (Figure 1). The Goldmann manual kinetic visual field was normal in both eyes. Red-green color vision defects were found in the Panel D15 and Ishihara tests. The ERG recordings showed a severely reduced but still recordable scotopic response and reduced combined and photopic responses with a decreased b-wave to a-wave ratio (Figure 3). Specialized ERGs using chromatic stimuli showed a reduced ML-mediated response and a supranormal S-cone-mediated response. In controls, the ML cone ERG amplitude is three times larger (a/b wave peaks) and 10 ms shorter (b-wave peak) than the S-cone ERG response. In this patient, the S-cone ERG presented a simplified waveform and a delayed and larger amplitude response as compared with normal controls and as compared with the ML-cone response (Figure 4).

Proband 2 was a 16 year old girl, referring visual disturbances since the age of 5. She was born from second cousins and her great grandmothers were sisters. No family history of ocular disorders was reported. At first examination, at the age of 6, her visual acuity was 0,0 LogMAR with a refractive error of +5.00 sph and +1.00 cyl/90 in the RE and 0.05 LogMAR with a refractive error of +6.00 sph and +1.00 cyl/90 in the LE; such functional values had remained stable over the years. A fundus examination showed a normal optic disk, regular vessel caliber, areas of chorioretinal atrophy with yellowish dots, and retinal pigment epithelium mottling at the posterior pole, more evident around the optic nerve and along the vascular arcades, and relatively preserved macular area. HD SD-OCT macular B-scans showed normal foveal morphology with intraretinal lacunae mainly in the ONL, bordering to the ELM, associated with smaller lacunae in the INL and focal hyper-reflectivity in the ganglion cell layer (GCL). Multiple areas of slightly irregular optical density without apparent fragmentation of ELM, the myoid zone, the EZ (ellipsoid), and outer segment photoreceptor layers were also present, mainly in the extrafoveal area.

**Figure 2:**
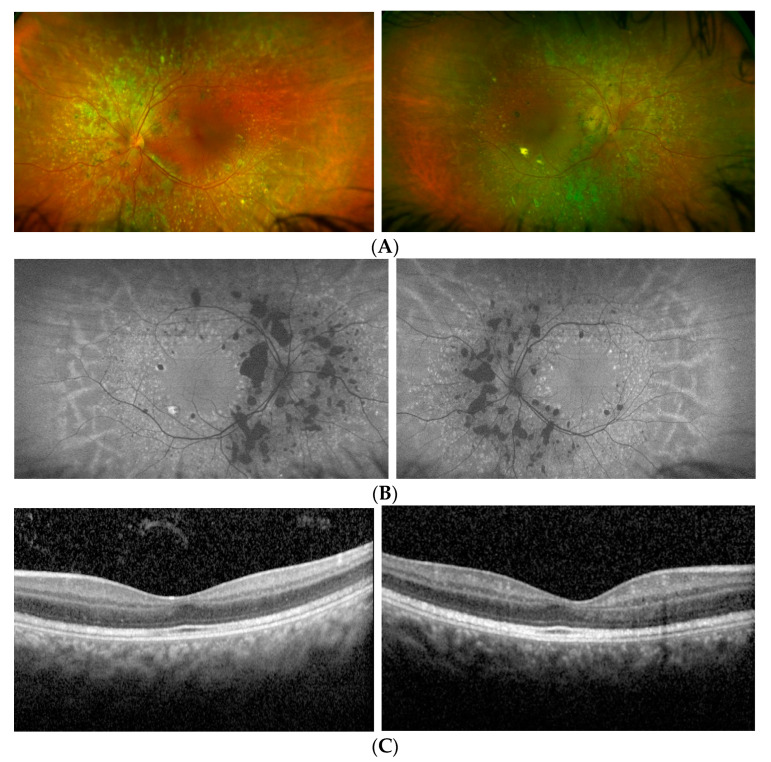
Retinal features of Patient 2: (**A**) Fundus of the right and left eye showing areas of chorioretinal atrophy with yellowish dots and retinal pigment epithelium mottling along the vascular arcades and relatively preserved macular area; (**B**) FAF images showing hypoautofluorescent patches corresponding to the atrophic lesions along the arcades and hyperautofluorescent spots corresponding to the yellowish dots; (**C**) HD SD-OCT macular B-scans showing normal foveal morphology with intraretinal lacunae mainly in the ONL, bordering to the ELM, associated with smaller lacunae in the INL and focal hyper-reflectivity in the GCL. The ELM, the myoid zone, the EZ, and the outer segment photoreceptor layers are not fragmented, but present multiple areas of slightly irregular optical density, mainly in the extrafoveal area.

**Figure 3:**
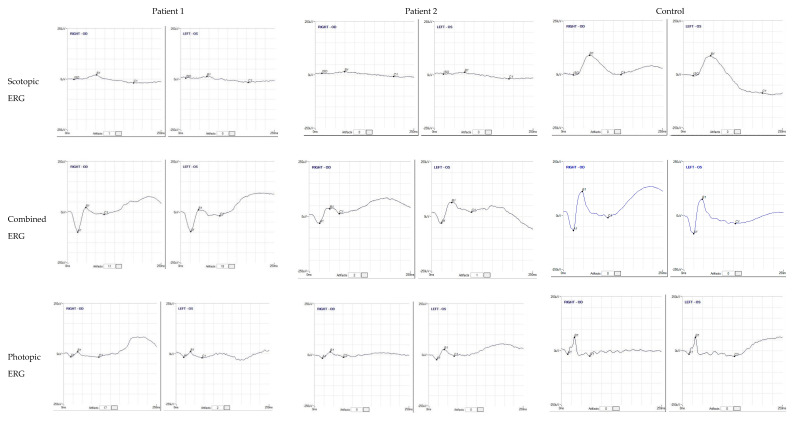
ERG responses. ERG recordings showing severely reduced responses for the scotopic stimulus and reduced responses for the combined and photopic stimuli with a decreased b-wave to a-wave ratio. Representative examples of ERG responses from a normal subject are shown for comparison.

**Figure 4:**
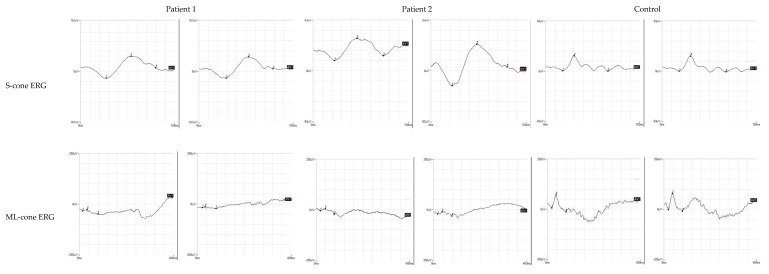
Specialized ERG responses. S-cone and ML-cone mediated ERG recordings from the patients and a control subject. Note that S-cone ERG is much larger than normal, delayed and with a simplified waveform as compared with the normal control.

FAF revealed extensive hypoautofluorescent patches corresponding to the atrophic lesions, while the yellow dots appeared hyperautofluorescent (Figure 2). The Goldmann manual kinetic visual field and color vision were normal. ERG recordings showed a severely reduced but still recordable scotopic response and reduced combined (with slight reduction of the b-wave to a-wave ratio) and photopic responses (Figure 3). Similarly to Patient 1, a specialized ERG recorded using chromatic stimuli showed a reduced ML-mediated response and a super-normal S-cone-mediated response. Again, S-cone ERG presented a simplified waveform and a delayed and larger amplitude response as compared with normal control values and as compared with ML-cone response (Figure 4). The findings found in the two patients indicated enhanced S-cone sensitivity and met the criteria for a clinical diagnosis of ESCS.

### 3.2. Genetic Analysis

The patients carried a novel frameshift homozygous variation in the *NRL* gene (NM_006177), c.22delC, resulting in the p.Leu8TrpfsTer11 change. This variant was confirmed in the heterozygous status in both parents and it was also validated by Sanger sequencing in the probands and their parents (see Supplementary Materials). The variation is not reported in gnomAD and was predicted to be damaging by in silico tools and assessed as pathogenic (class 5) according to ACMG criteria. The identified variation determines a premature stop codon leading to the putative complete lack of the protein. The p.Leu8TrpfsTer11 variant has not been previously described. A variant in the same codon, c.23delT (p.Leu8ArgfsTer11), was reported in heterozygous status in a study of a large cohort of RP and Usher syndrome patients, even if no functional or segregation analysis had been performed [23].

## 4. Discussion

Enhanced S-cone syndrome (ESCS) is a very rare disease mainly associated with variants in the *NR2E3* gene which has been reported to account for up to 93% of cases [24]. The *NRL* gene plays an essential role, together with the *NR2E3* gene, in inducing photoreceptor precursors to a rod fate and suppressing a cone fate. Most missense variations in the *NRL* gene are dominant and have been reported to cause RP (Table 1), while retinal dystrophies caused by autosomal recessive *NRL* variants are scarce. So far, only seven cases of autosomal recessive *NRL*-associated ESCS have been described. Nishigushi et al. suggested an ESCS phenotype in two siblings who presented the typical clumped pigmentary retinal degeneration and relative preservation of blue cone function associated with compound heterozygous *NRL* variations: a loss-of-function (c.224_225insC, p.Ala76GlyfsTer18) and a missense variant (c.479T>C, p.Leu160Pro) [14]. Newman et al. reported on two unrelated patients, also affected by oculopharyngeal muscular dystrophy, with a ESCS-like phenotype that confirmed the predominance of S-cones using S-cone-specific ERG measurements which showed the typical delayed waveform to white light stimulus in dark-adapted as well as light-adapted responses. Genetic analysis revealed a novel homozygous loss-of-function variation located in exon 3, leading to the substitution of a codon for arginine by a stop codon at position 31 of the NRL protein (c.91C>T; p.Arg31Ter) [15]. Three Moroccan patients from two different families who presented a reduced visual acuity associated with typical clumped pigmentary retinal degeneration were described by Littink et al. One patient carried a homozygous missense variant (c.508C>A; p.Arg170Ser) in the *NRL* gene, whereas the same change was identified heterozygously in the two siblings of a second family, associated with a one base pair deletion (c.654del; p.Cys219ValfsTer4) on the other allele [19]. In a case series report on 27 patients with confirmed ESCS by ERG recording, only one patient carried a heterozygous *NRL* insertion (c.223insC; p.Leu75fsProTer18). Since a second variant was not found and the same variant was present heterozygously in unaffected family members, the authors suggested a digenic mechanism with another unknown gene [24]. However, we could hypothesize that a second heterozygous null pathogenic variation in *NRL*, such as a large deletion or duplication or a deep intronic variant or an alteration in a regulatory site was not detected at the time. *NRL* has been suggested as a likely candidate gene for ESCS, as studies with ERG on *Nrl*−/− mouse have shown a complete loss of rod function and supranormal blue cone function [15]. Such functional evidence has been confirmed by morphological, immunohistochemical, and gene-expression analyses, demonstrating the presence of S-cone immunoreactivity and an increased S-cone-specific gene expression. Noteworthy, it was recently demonstrated that *NRL*-Leu75ProfsTer19-derived human retinal organoids, constructed via an in vitro human stem cell-based system in order to examine photoreceptor development, displayed a phenotype similar to that of an *Nrl* null mouse [25]. Furthermore, rods were not detected by immunocytochemistry and the portion of the outer nuclear layer normally populated by rods in wild type organoids contained S-opsin+ photoreceptors. The authors indicated that this defect of the photoreceptors ultimate determination was accompanied by an evident alteration in the S- to ML-opsin+ photoreceptors ratio. Taken together, these data may suggest that the *NRL* c.223dupC mutation could potentially lead to ESCS in humans. Common clinical features of *NRL*-related ESCS so far described are optic disk pallor, clumps of pigment in the periphery at the level of retinal pigment epithelium with shapes that are distinct from the bone spicule-shaped pigment deposits seen in patients with typical RP, the presence of white dots mainly in the peripheral retina, cystoid maculopathy, and various stages of atrophic retinal pigment epithelium changes in the macula [14,15,16]. Peculiar responses of ERGs in ESCS are represented by enhanced short-wavelength sensitivity and absent rod function.

In the present report, we described two unrelated pediatric patients with a novel recessive loss-of-function *NRL* variation. An extensive morpho-functional analysis led us to the diagnosis of ESCS. Interestingly, the two patients, despite having the same mutation, showed a relatively different phenotype. Patient 1 presented an early onset disease characterized by reduced visual acuity, nystagmus, and marked photophobia, with funduscopic alterations mainly affecting the posterior pole along the arcades and the peripheral retina. The OCT macular scans showed macular cysts, which were greater in the central fovea and affected different retinal layers associated with a significant alteration of the outer photoreceptor layer. These changes of the OCT substructure are consistent with diffuse photoreceptoral damage, involving both the cone (foveal changes) and rods (extrafoveal changes). The intraretinal lacunae and microcysts in the ONL could be related to a remodeling of the inner retinal cells contiguous to the focally altered photoreceptor layers. Conversely, Patient 2, although presenting early-onset visual disturbances, maintained a relatively preserved visual acuity over the years with more severe peripheral retinal alteration as compared with Patient 1, consisting of extensive areas of chorioretinal atrophy with yellowish dots and retinal pigment epithelium mottling, mainly peripapillary and along the vascular arcades and sparing of the macular area. The OCT macular scans showed normal foveal morphology with small perifoveal microcysts at the level of the INL and ONL and minimal changes in the optical density of the photoreceptoral layer, more in the extrafoveal area, thus, expressing mainly rod damage with relatively preserved foveal cones. Vice versa, the ERG recordings showed a similar pattern in the two patients with a severely reduced, but still recordable, scotopic response and reduced combined, with a decreased b-wave to a-wave ratio, and photopic responses. Similarly, the ERG recordings elicited from chromatic stimuli showed a relatively homogeneous pattern, consistent with the clinical diagnosis of ESCS and characterized by a reduced ML-mediated response and a super-normal S-cone mediated response with simplified waveform. Since early childhood, both patients had presented signs of visual impairment but the course of the disease was slowly progressive in Patient 1 and relatively stable in Patient 2. Visual acuity had been significantly reduced since the onset of the disease, slowly decreasing over the years in Patient 1, maybe as the result of the cystoid maculopathy, whereas Patient 2 showed a relatively good and, thus far, stable level of vision maintaining a preserved foveal morphology. The differences in the clinical expression of the disease observed in our two patients are in accordance with previous reports describing the variability of clinical expression in *NR2E3*-associated ESCS with funduscopic features ranging from normal to severely altered with marked pigmentary changes [23,24,38]. Describing a group of children affected by *NR2E3*-associated ESCS, Hull et al. hypothesized a progressive change from an apparent normal fundus followed by retinal pigment epithelium mottling along the arcades, development of white dots, and, finally, deep nummular pigmentary deposition [38]. No patients with nystagmus were reported, whereas one of our patients was affected, suggesting a younger age of onset or a more severe presentation in some cases of *NRL*-associated ESCS as compared with *NR2E3*-related ESCS. Moreover, the nearly similar age of our two patients at the observational time, presenting a different course of the disease with a significant visual dysfunction and macular alteration in one case and a severely affected peripheral retina with relatively preserved visual acuity and macular morphology in the other case, may suggest a more variable clinical expression of the *NRL* mutation, perhaps due to unknown regulating factors, than a progressive sequence of phenotypic changes. This was in accordance with a previous report of patients that showed only subtle pigmentary alterations up to an elderly age [39]. Nevertheless, the few cases of *NRL*-associated ESCS described to date preclude making an extensive comparative analysis between the clinical expression of the two causative genes. Additionally, another frequently reported homologous clinical sign is the presence of elevated hyperopia in *NR2E3*-associated ESCS and *NRL*-associated ESCS. This evidence, although common to other congenital and early-onset retinal dystrophies, might suggest problems with emmetropization during development.

In conclusion, this report expands the clinical and genetic spectrum of ESCS due to *NRL* recessive inheritance, supporting its disease causative role, and leads to confirming the variability of clinical presentation observed in *NR2E3*-associated ESCS.

## Figures and Tables

**Table 1:** Known pathogenic variations in the *NRL* gene.

Known Pathogenic Variations in *NRL* Gene (NM_006177)			
**Missense/Nonsense Variations**		**Phenotype**	**Ref.**
**DNA Change**	**Protein Change**		
c.91C>T	p.(Arg31Ter)	Night blindness and reduced visual acuity, *Autosomal Recessive*	[15]
c.146C>T	p.(Pro49Leu)	Retinitis pigmentosa, *Autosomal Dominant*	[11]
c.148T>A	p.(Ser50Thr)	Retinitis pigmentosa, *Autosomal Dominant*	[9]
c.148T>C	p.(Ser50Pro)	Retinitis pigmentosa, *Autosomal Dominant*	[10]
c.149C>T	p.(Ser50Leu)	Retinitis pigmentosa, *Autosomal Dominant*	[10]
c.151C>A	p.(Pro51Thr)	Retinitis pigmentosa, *Autosomal Dominant*	[10]
c.151C>G	p.(Pro51Ala)	Retinitis pigmentosa, *Autosomal Dominant*	[26]
c.151C>T	p.(Pro51Ser)	Retinitis pigmentosa, *Autosomal Dominant*	[14]
c.152C>T	p.(Pro51Leu)	Retinitis pigmentosa, *Autosomal Dominant*	[27]
c.152C>G	p.(Pro51Arg)	Rod cone dystrophy, *unclear mode of transmission*	[28]
c.287T>C	p.(Met96Thr)	Retinitis pigmentosa, *Autosomal Dominant*	[12]
c.238C>T	p.(Gln80Ter)	Retinitis pigmentosa, *Autosomal Recessive*	[29]
c.339C>G	p.(Tyr113Ter)	Goldmann-Favre syndrome, *Autosomal Recessive*	[30]
c.365G>A	p.(Gly122Glu)	Retinitis pigmentosa, *Autosomal Dominant*	[27]
c.416C>G	p.(Ser139Trp)	Leber congenital amaurosis, *Autosomal Recessive*	[31]
c.424G>A	p.(Val142Met)	Retinitis pigmentosa, *Autosomal Dominant*	[13]
c.479T>C	p.(Leu160Pro)	Clumped pigmentary retinal degeneration, *Autosomal Recessive*	[14]
c.508C>A	p.(Arg170Ser)	Clumped pigmentary retinal degeneration, *Autosomal Recessive*	[16,19]
c.520C>A	p.(Gln174Lys)	Leber congenital amaurosis/ retinal dystrophy, *Autosomal Recessive*	[32]
c.544C>T	p.(Gln182Ter)	Retinitis pigmentosa, *Autosomal Recessive*	[29]
c.674G>A	p.(Ser225Asn)	Cone dysfunction syndrome, *uncertain inheritance pattern*	[14]
c.713G>T	p.(Ter238Leu)	Retinitis pigmentosa, *Autosomal Recessive*	[29]

**Indel Variations**		**Phenotype**	**Ref.**
**DNA Change**	**Protein Change**		
c.16delA	p.(Ser6AlafsTer13)	Leber congenital amaurosis, atypical, *Autosomal Recessive*	[33]
c.23delT	p.(Leu8ArgfsTer11)	Retinitis pigmentosa, *Autosomal Dominant*	[23]
c.104dup	p.(Thr36fs)	Retinitis pigmentosa, *Autosomal Recessive*	[34]
c.147_149delTTC	p.(Ser50del)	Retinitis pigmentosa, *Autosomal Dominant*	[35]
c.223dupC	p.(Leu75ProfsTer19)	Chorioretinal Dystrophy, *Autosomal Recessive*	[36]
c.223insC	p.(Leu75fsProTer18)	Enhanced S-cone syndrome *uncertain inheritance pattern*	[24]
c.224_225insC	p.(Leu75fsTer)	Clumped pigmentary retinal degeneration, *Autosomal Recessive*	[14,24]
c.386delC	p.(Ala129GlufsTer17)	Leber congenital amaurosis, atypical, *Autosomal Recessive*	[33]
c.444_445insGCTGCGGG	p.(Leu149AlafsTer15)	Retinitis pigmentosa, *Autosomal Recessive*	[18]
c.452_459dupGCTGCGGG	p.(Arg154AlafsTer10)	Retinitis pigmentosa, *Autosomal Recessive*	[18]
c.586_627dupGCCCAGCTGGACGCGCTGCGGGCCGAGGTGGCCCGCCTGGCC	p.(Ala196_Ala209dup)	Retinal disease, *Autosomal Dominant*	[37]
c.654delC	p.(Cys219ValfsTer4)	Leber congenital amaurosis, *Autosomal Recessive*	[14,19]

## Data Availability

For more detailed information on the genetic methodology and process used for this study, please contact Lorenzo Sinibaldi (lorenzo.sinibaldi@opbg.net) and Chiara Passarelli (chiara.passarelli@opbg.net).

## Supplementary Materials

The following supporting information can be downloaded at: https://www.mdpi.com/article/10.3390/diagnostics12092183/s1.

## References

[1] Vincent A., Robson A.G., Holder G.E. (2013). Pathognomonic (diagnostic) ERGs A Review and Update. Retina.

[2] Jacobson S.G., Marmor M.F., Kemp C.M., Knighton R.W. (1990). SWS (blue) cone hypersensitivity in a newly identified retinal de-generation. Investig. Ophthalmol. Vis. Sci..

[3] Marmor M.F., Jacobson S.G., Foerster M.H., Kellner U., Weleber R.G. (1990). Diagnostic Clinical Findings of a New Syndrome with Night Blindness, Maculopathy, and Enhanced S Cone Sensitivity. Am. J. Ophthalmol..

[4] Cheng H., Khanna H., Oh E.C., Hicks D., Mitton K., Swaroop A. (2004). Photoreceptor-specific nuclear receptor NR2E3 functions as a transcriptional activator in rod photoreceptors. Hum. Mol. Genet..

[5] Kanda A., Friedman J.S., Nishiguchi K.M., Swaroop A. (2007). Retinopathy mutations in the bZIP protein NRL alter phosphoryla-tion and transcriptional activity. Hum. Mutat..

[6] Farjo Q., Jackson A., Pieke-Dahl S., Scott K., Kimberling W.J., Sieving P.A., Richards J.E., Swaroop A. (1997). Human bZIP Transcription Factor GeneNRL:Structure, Genomic Sequence, and Fine Linkage Mapping at 14q11.2 and Negative Mutation Analysis in Patients with Retinal Degeneration. Genomics.

[7] Swaroop A., Xu J.Z., Pawar H., Jackson A., Skolnick C., Agarwal N. (1992). A conserved retina-specific gene encodes a basic motif/leucine zipper domain. Proc. Natl. Acad. Sci. USA.

[8] Mears A.J., Kondo M., Swain P.K., Takada Y., Bush R.A., Saunders T.L., Sieving P.A., Swaroop A. (2001). NRL is required for rod photoreceptor development. Nat. Genet..

[9] Bessant D.A., Payne A.M., Mitton K.P., Wang Q.-L., Swain P.K., Plant C., Bird A.C., Zack D.J., Swaroop A., Bhattacharya S.S. (1999). A mutation in NRL is associated with autosomal dominant retinitis pigmentosa. Nat. Genet..

[10] DeAngelis M.M., Grimsby J.L., Sandberg M.A., Berson E.L., Dryja T.P. (2002). Novel mutations in the NRL gene and associated clinical findings in patients with dominant retinitis pigmentosa. Arch. Ophthalmol..

[11] Gao M., Zhang S., Liu C., Qin Y., Archacki S., Jin L., Wang Y., Liu F., Chen J., Liu Y. (2016). Whole exome sequencing identifies a novel NRL mutation in a Chinese family with autosomal dominant retinitis pigmentosa. Mol. Vis..

[12] Hernan I., Gamundi M.J., Borràs E., Maseras M., García-Sandoval B., Blanco-Kelly F., Ayuso C., Carballo M. (2012). Novel p.M96T variant of NRL and shRNA-based suppression and replacement of NRL mutants associated with autosomal dominant retinitis pig-mentosa. Clin. Genet..

[13] Gao F.-J., Li J.-K., Chen H., Hu F.-Y., Zhang S.-H., Qi Y.-H., Xu P., Wang D.-D., Wang L.-S., Chang Q. (2019). Genetic and Clinical Findings in a Large Cohort of Chinese Patients with Suspected Retinitis Pigmentosa. Ophthalmology.

[14] Nishiguchi K.M., Friedman J.S., Sandberg M.A., Swaroop A., Berson E.L., Dryja T.P. (2004). Recessive *NRL* mutations in patients with clumped pigmentary retinal degeneration and relative preservation of blue cone function. Proc. Natl. Acad. Sci. USA.

[15] Newman H., Blumen S.C., Braverman I., Hanna R., Tiosano B., Perlman I., Ben-Yosef T. (2016). Homozygosity for a Recessive Loss-of-Function Mutation of the *NRL* Gene Is Associated With a Variant of Enhanced S-Cone Syndrome. Investig. Opthalmology Vis. Sci..

[16] Collin R.W.J., Born L.I.V.D., Klevering B.J., de Castro-Miró M., Littink K.W., Arimadyo K., Azam M., Yazar V., Zonneveld M.N., Paun C.C. (2011). High-Resolution Homozygosity Mapping Is a Powerful Tool to Detect Novel Mutations Causative of Autosomal Recessive RP in the Dutch Population. Investig. Opthalmology Vis. Sci..

[17] Neveling K., Collin R.W., Gilissen C., van Huet R.A., Visser L., Kwint M.P., Gijsen S.J., Zonneveld M.N., Wieskamp N., de Ligt J. (2012). Next-generation genetic testing for retinitis pigmentosa. Hum. Mutat..

[18] Beryozkin A., Shevah E., Kimchi A., Mizrahi-Meissonnier L., Khateb S., Ratnapriya R., Lazar C.H., Blumenfeld A., Ben-Yosef T., Hemo Y. (2015). Whole Exome Sequencing Reveals Mutations in Known Retinal Disease Genes in 33 out of 68 Israeli Families with Inherited Retinopathies. Sci. Rep..

[19] Littink K.W., Stappers P., Riemslag F., Talsma H.E., Van Genderen M.M., Cremers F., Collin R., Van den Born L.I. (2018). Autosomal recessive NRL mutations in patients with enhanced Scone syndrome. Genes.

[20] Sustar M., Hawlina M., Brecelj J. (2011). Electroretinographic evaluation of the retinal S-cone system. Doc. Ophthalmol..

[21] Kopanos C., Tsiolkas V., Kouris A., Chapple C.E., Aguilera M.A., Meyer R., Massouras A. (2019). VarSome: The human genomic variant search engine. Bioinformatics.

[22] Richards S., Aziz N., Bale S., Bick D., Das S., Gastier-Foster J., Grody W.W., Hegde M., Lyon E., Spector E. (2015). ACMG Laboratory Quality Assurance Committee. Standards and guidelines for the interpretation of sequence variants: A joint consensus rec-ommendation of the American College of Medical Genetics and Genomics and the Association for Molecular Pathology. Genet. Med..

[23] Oishi M., Oishi A., Gotoh N., Ogino K., Higasa K., Iida K., Makiyama Y., Morooka S., Matsuda F., Yoshimura N. (2014). Comprehensive molecular diagnosis of a large cohort of Japanese retinitis pigmentosa and Usher syndrome patients by next-generation se-quencing. Investig. Ophthalmol. Vis. Sci..

[24] Wright A.F., Reddick A.C., Schwartz S.B., Ferguson J.S., Aleman T.S., Kellner U., Jurklies B., Schuster A., Zrenner E., Wissinger B. (2004). Mutation analysis of NR2E3 and NRL genes in Enhanced S Cone Syndrome. Hum. Mutat..

[25] Kallman A., Capowski E.E., Wang J., Kaushik A.M., Jansen A.D., Edwards K.L., Chen L., Berlinicke C.A., Phillips M.J., Pierce E.A. (2020). Investigating cone photoreceptor development using patient-derived NRL null retinal organoids. Commun. Biol..

[26] Daiger S.P., Bowne S.J., Sullivan L.S., Blanton S.H., Weinstock G.M., Koboldt D.C., Fulton R.S., Larsen D., Humphries P., Humphries M.M. (2014). Application of next-generation sequencing to identify genes and mutations causing autosomal dominant retinitis pigmentosa (adRP). Single Mol. Single Cell Seq..

[27] Martinez-Gimeno M., Maseras M., Baiget M., Beneito M., Antiñolo G., Ayuso C., Carballo M. (2001). Mutations P51U and G122E in retinal transcription factor NRL associated with autosomal dominant and sporadic retinitis pigmentosa. Hum. Mutat..

[28] Hull S., Kiray G., Chiang J.P., Vincent A.L. (2020). Molecular and phenotypic investigation of a New Zealand cohort of childhood-onset retinal dystrophy. Am. J. Med. Genet. Part C Semin. Med. Genet..

[29] El-Asrag M.E., Corton M., McKibbin M., Avila-Fernandez A., Mohamed M.D., Blanco-Kelly F., Toomes C., Inglehearn C.F., Ayuso C., Ali M. (2022). Novel homozygous mutations in the transcription factor NRL cause non-syndromic retinitis pigmentosa. Mol. Vis..

[30] De Castro-Miró M., Tonda R., Escudero-Ferruz P., Andrés R., Mayor-Lorenzo A., Castro J., Ciccioli M., Hidalgo D.A., Rodríguez-Ezcurra J.J., Farrando J. (2016). Novel Candidate Genes and a Wide Spectrum of Structural and Point Mutations Responsible for Inherited Retinal Dystrophies Revealed by Exome Se-quencing. PLoS ONE.

[31] Porto F.B.O., Jones E.M., Branch J., Soens Z.T., Maia I.M., Sena I.F.G., Sampaio S.A.M., Simões R.T., Chen R. (2017). Molecular Screening of 43 Brazilian Families Diagnosed with Leber Congenital Amaurosis or Early-Onset Severe Retinal Dystrophy. Genes.

[32] Xu K., Xie Y., Sun T., Zhang X., Chen C., Li Y. (2020). Genetic and clinical findings in a Chinese cohort with Leber congenital amaurosis and early onset severe retinal dystrophy. Br. J. Ophthalmol..

[33] Carrigan M., Duignan E., Malone C., Stephenson K., Saad T., McDermott C., Green A., Keegan D., Humphries P., Kenna P.F. (2016). Panel-Based Population Next-Generation Sequencing for Inherited Retinal Degenerations. Sci. Rep..

[34] Haer-Wigman L., Van Zelst-Stams W.A.G., Pfundt R., Born L.I.V.D., Klaver C., Verheij J.B.G.M., Hoyng C.B., Breuning M.H., Boon C., Kievit A.J. (2017). Diagnostic exome sequencing in 266 Dutch patients with visual impairment. Eur. J. Hum. Genet..

[35] Qin Y., Liu F., Yu S., Yang L., Gao M., Tang Z., Guo A.Y., Zhang M., Li P., Liu M. (2017). Identification of a novel NRL mutation in a Chinese family with retinitis pigmentosa by whole-exome sequencing. Eye.

[36] Consugar M.B., Navarro-Gomez D., Place E.M., Bujakowska K.M., Sousa M.E., Fonseca-Kelly Z.D., Taub D.G., Janessian M., Wang D.Y., Au E.D. (2014). Panel-based genetic diagnostic testing for inherited eye diseases is highly accurate and reproducible, and more sensitive for variant detection, than exome sequencing. Genet. Med..

[37] Ellingford J.M., Barton S., Bhaskar S., O’Sullivan J., Williams S.G., Lamb J.A., Panda B., Sergouniotis P.I., Gillespie R.L., Daiger S.P. (2016). Molecular findings from 537 individuals with inherited retinal disease. J. Med. Genet..

[38] Hull S., Arno G., Sergouniotis P.I., Tiffin P., Borman A.D., Chandra A., Robson A., Holder G.E., Webster A.R., Moore A.T. (2014). Clinical and Molecular Characterization of Enhanced S-Cone Syndrome in Children. JAMA Ophthalmol..

[39] Audo I., Michaelides M., Robson A.G., Hawlina M., Vaclavik V., Sandbach J.M., Neveu M.M., Hogg C.R., Hunt D.M., Moore A.T. (2008). Phenotypic Variation in Enhanced S-cone Syndrome. Investig. Opthalmology Vis. Sci..

