# Supplementary material for: A Novel Autosomal Recessive Variant of the NRL Gene Causing Enhanced S-Cone Syndrome: A Morpho-Functional Analysis of Two Unrelated Pediatric Patients
Source: Diagnostics (Basel). 2022 Sep 9;12(9):2183. doi: 10.3390/diagnostics12092183 (PMC9497687; doi:10.3390/diagnostics12092183)
Supplement: Supplementary file 1 [file diagnostics-12-02183-s001.zip › diagnostics-1912469-supplementary.pdf]

Supplemental Material

A - Pedigree of Patient 1

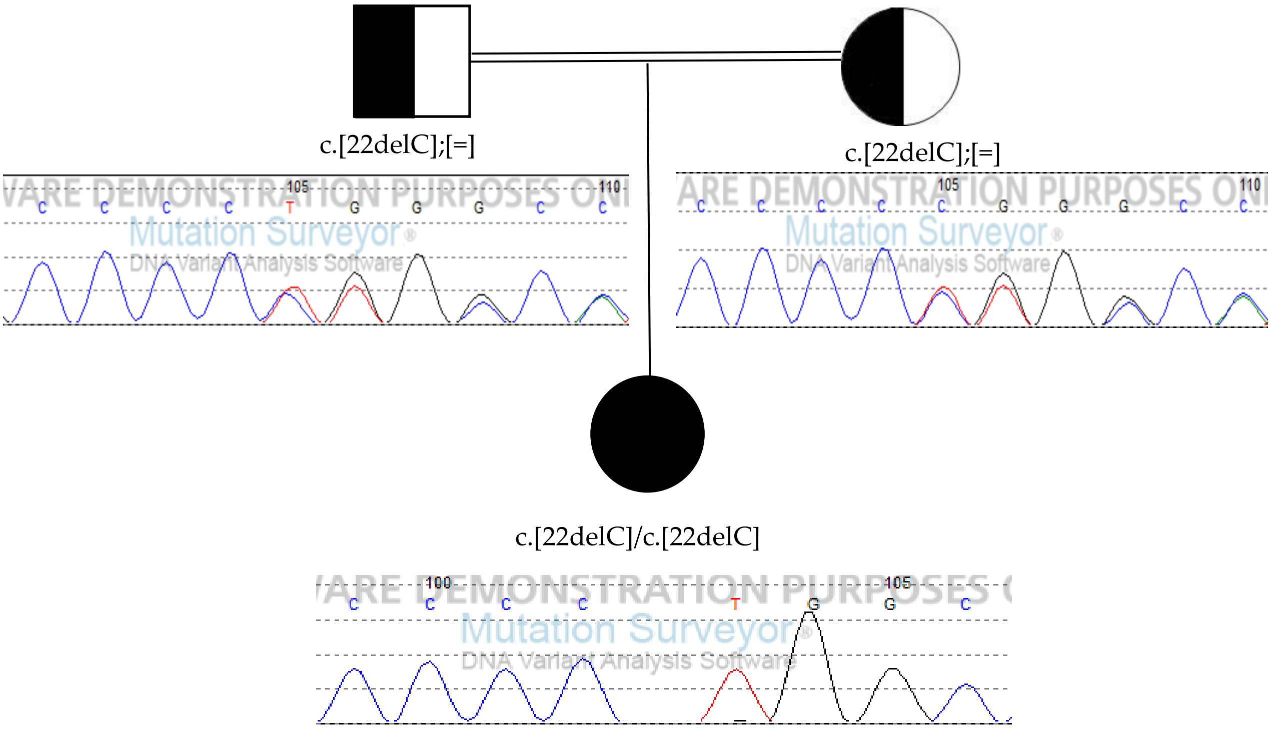

B - Pedigree of Patient 2

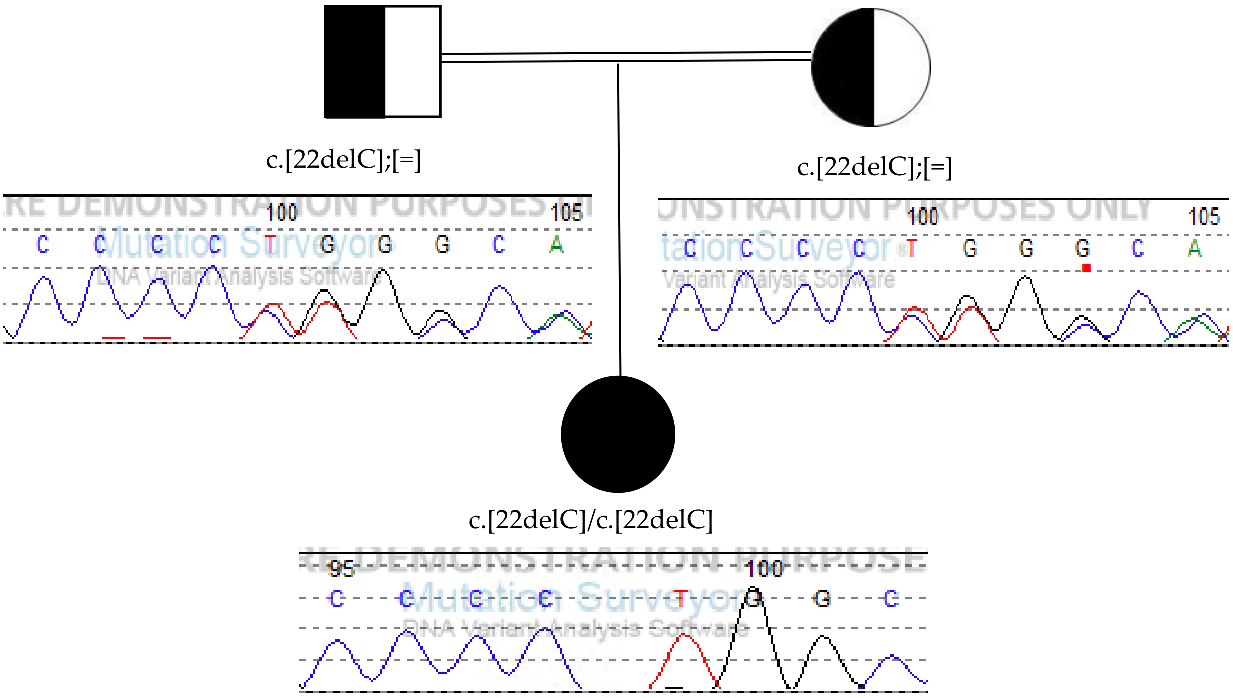

FIGURE LEGEND: Pedigrees of families of patient 1 (A) and patient 2 (B) with the variant in *NRL* gene in homozygous status in both patients and heterozygous status in the parents, confirmed by Sanger sequencing.
